# Role of 99mTc-HIDA Scan for Assessment of Gallbladder Dyskinesia and Comparison of Gallbladder Dyskinesia with Various Parameters in Laparoscopic Cholecystectomy Patients

**DOI:** 10.1155/2019/5705039

**Published:** 2019-02-14

**Authors:** Manuneethimaran Thiyagarajan, Eniyan Kamaraj, Nitesh Navrathan, Mohanapriya Thyagarajan, Balaji Singh Krishna

**Affiliations:** General Surgery Department, Sri Ramachandra Medical University, Chennai, India

## Abstract

**Objectives:**

Pathogenesis of gallstone includes bile stasis due to defect in the gallbladder muscle contraction. Our aim of the study is to find out the role of 99mTc-HIDA scan in assessment of gallbladder dyskinesia in cholelithiasis patients before laparoscopic cholecystectomy and compare the gallbladder dyskinesia with various parameters like symptoms of patients, diabetic status of patients, gallstones size and number, and cholecystitis features in histopathology report after surgery.

**Material and Method:**

This is a prospective observational study conducted at our hospital for three years. Totally 40 patients with gallstone were subjected to 99mTc-HIDA scan, to assess the ejection fraction of gallbladder. For all these patients detailed clinical history, presence of comorbid illness like diabetics, and symptomatology were elicited. For all patients, ultrasonogram of abdomen was done to assess number and size of stones. All parameters were tabulated and correlated.

**Result:**

While comparing 99mTc-HIDA scan findings with symptoms of patients, 21.2% were asymptomatic and 78.8% symptomatic patients who had ejection fraction less than 80%. All patients in EF >80% group were symptomatic only. It is not statistically significant. On comparing 99mTc-HIDA scan findings with diabetic status of the patients, 42.4% of diabetic and 57.6 % of nondiabetic patients had ejection fraction less than 80%. It is not statistically significant (0.681). While comparing 99mTc-HIDA scan findings with size of the gallstone in ultrasound, 63.6% patients with size less than 1cm and 36.4% with size more than 1cm had ejection fraction < 80%. It is statistically significant (0.048). On comparing 99mTc-HIDA scan findings with number of stones in ultrasound, 18.2% single gallstone patients and 81.8% multiple gallstone patients had EF less than 80% which is statistically significant (0.001). While comparing the 99mTc-HIDA scan findings with histopathology report after laparoscopic cholecystectomy, 21.2% non-cholecystitis patients and 78.8% cholecystitis patients had EF less than 80%, which is statistically (0.017) significant.

**Conclusion:**

99mTc-HIDA scan can be an accurate method to diagnose the gallbladder dyskinesia. Gallbladder dyskinesia in 99mTc-HIDA scan can be used to predict large size stones and multiple stones before surgery. The sensitivity can be improved by 99mTc-HIDA scan in diagnosing cholecystitis patients.

## 1. Introduction

Bile storage in the gallbladder changes according to digestive and interdigestive phases. It is stored in the gallbladder in interdigestive phase and emptied during digestive phase. 90% of bile diversion from liver to gallbladder is based on the motility of gallbladder and sphincter of Oddi and only 10% of bile directly drains from liver to duodenum [1]. However during the interdigestive and digestive periods, gallbladder emptying and bile refilling into gallbladder occur [2, 3].

Relaxation of sphincter of Oddi determines the free flow of bile from gallbladder into duodenum. A defect in the sphincter mechanism results in bile stasis and leads to gallstone formation [4].

In pregnancy high circulating levels of progesterone lead to defective muscle contraction of gallbladder. Supersaturation of bile with cholesterol also causes gallbladder dyskinesia. This dyskinesia of gallbladder is one of the commonest causes for acute cholecystitis and chronic cholecystitis. In addition, recurrent biliary pain in acalculous gallbladder patients may be because of this impaired gallbladder contraction [5].

In gallbladder disease patients, the standard investigation to diagnose gallstone is transabdominal ultrasonography (TUS)[6]. Patients with typical biliary pain are often underinvestigated by TUS scan alone. There is a false negative rate of 2 –5%, with reduced sensitivity in TUS for detection of very small gallstones and common bile duct stones [7, 8]. Chronic irritation and inflammation of gallbladder wall can be a result of bile stasis due to gallbladder dysmotility. Whipple in 1922 first described the benefit of cholecystectomy for acalculous biliary colic patients on long-term follow-up. He reported 76% symptom resolution in these patients after cholecystectomy [9].

The other options for further investigation in biliary symptom patients with normal TUS include endoscopic ultrasonography and radionucleotide scanning. For structural abnormalities such as gallbladder sludge, microlithiasis, and choledocholithiasis, diagnostic sensitivity increased by endoscopic ultrasonography over transabdominal scanning. The assessment of gallbladder function well diagnosed with the provocative oral cholecystogram [10] and more recently provocative radionuclide cholescintigraphy is used as a preferred method to assess the gallbladder function [11–15]. Although 99m technetium-labeled hepatoiminodiacetic acid (99mTc-HIDA) radionuclide scanning has been available for over a decade, it is not used routinely as diagnostic tool in patients with biliary symptoms [11, 12]. Most units preferred provocative scan (99mTc-HIDA) using cholecystokinin octapeptide infusion. The rate of infusion varies in different published literature but it is preferred to be from 3 to 30 min by various authors [16–18].

In patients with biliary pain and with normal TUS scan, 99mTc-HIDA scan is a useful diagnostic tool [19].

## 2. Aim and Objective

Our aim of the study is to find out the role of 99mTc-HIDA scan in assessment of gallbladder dyskinesia in cholelithiasis patients before laparoscopic cholecystectomy and compare the gallbladder dyskinesia with various parameters like symptoms of patients, diabetic status of patients, gallstone size, number of stones, and cholecystitis features in histopathology report after surgery.

## 3. Materials and Methods

This is a prospective observational study conducted in our hospital from 2015 to 2018. Totally 40 patients with gallstone disease were included in this study. For all these patients detailed clinical history, presence of comorbid illness like diabetics, and symptomatology were elicited. For all patients ultrasonogram of abdomen was done to assess number of stones and size of stones. Also all the patients were subjected to 99mTc-HIDA scan, to assess the ejection fraction of gallbladder. All parameters were tabulated and correlated. Ejection fraction more than 80% was taken as normal and less than 80% counted as gallbladder dyskinesia.

First patients were divided as diabetic and nondiabetic patients. Gallbladder contractility in 99mTc-HIDA scan was compared in diabetic versus nondiabetic patients. Then patients were divided as symptomatic patients and asymptomatic gallstone patients and compared in terms of gallbladder contractility in 99mTc-HIDA scan by software analysis. Based on ultra-scan abdomen patients were grouped into single stone patients and multiple gallstone patients and compared in terms of gallbladder contractility. Patients were also grouped by size of the stone in ultrasonogram. Gallstone size more than 1cm and gallstone size less than and equal to 1cm were grouped separately. Comparison of gallbladder contractility was done with this group with software analysis. After laparoscopic cholecystectomy was done, histopathology reports of the gallbladder specimen were collected. In histopathology report cholecystitis features were grouped separately. In histopathology report any features other than cholecystitis were grouped separately. We have compared the gallbladder contractility in 99mTc-HIDA scan with these groups by software analysis.

## 4. Software Analysis

The collected data were analyzed with IBM.SPSS statistics software version 23.0. For the data descriptive statistics frequency analysis, percentage analysis was used for categorical variables and the mean & SD were used for continuous variables.

To find the significant difference between the bivariate sample in independent groups the unpaired sample, t -test was used. In the above statistical tool the probability value less than* 0.05* is considered as significant level.

## 5. Observation and Result

This is a prospective study in cholelithiasis patients, comparing gallbladder ejection fraction with various parameters of the patients. Out of 40 patients included in this study 62.5% (25) were females and 37.5% (15) were males

Table 1 shows the result of comparison of 99mTc-HIDA scan report with symptomatic versus asymptomatic patients, diabetic versus nondiabetic patients, gallstone size >1cm versus less than and equal to 1cm patients, multiple versus single stone patients, and cholecystitis versus no cholecystitis in HPE report.

While comparing 99mTc-HIDA scans findings with symptoms of patients 21.2% were asymptomatic and 78.8% symptomatic patients who had ejection fraction less than 80%. All patients in EF >80% groups were symptomatic only. The P value obtained using Chi-Square test (0.317 ) is insignificant. The commonest symptom is abdominal pain (82.5%) followed by fever (22.5%) and dyspepsia (17.5%).

When comparing 99mTc-HIDA scan findings with diabetic status of the patients, 42.4% of diabetic and 57.6 % of nondiabetic patients had ejection fraction less than 80%, whereas 28.6% of diabetic and 71.4% of nondiabetic patients had EF >80%. The P value obtained using Chi-Square test (0.681) is insignificant.

While comparing 99mTc-HIDA scan findings with size of the gallstone in ultrasound, 63.6% patients with size less than 1cm and 36.4% with size more than 1cm had ejection fraction < 80%. Among the patients included in the study who had ejection fraction more than 80% no patient had size more than 1cm stone. The P value obtained using Chi-Square test is (0.048) significant.

While comparing 99mTc-HIDA scan findings with number of stones in ultrasound, 18.2% single gallstone patients and 81.8% multiple gallstones patients had EF less than 80%. Furthermore 85.7% single stone patients and 14.3% multiple gallstones patients had EF more than 80%. The P value obtained using Chi-Square test is (0.001) significant.

While comparing the 99mTc-HIDA scan findings with histopathology report after laparoscopic cholecystectomy, 21.2% non-cholecystitis patients and 78.8% cholecystitis patients had EF less than 80%. But 71.4% non-cholecystitis patients and 28.6% cholecystitis patients had EF more than 80%. The P value obtained using Chi-Square test is (0.017) significant.

## 6. Discussion

The gallbladder dyskinesia is diagnosed by performing gallbladder ejection fraction There are various methods to study the gallbladder contractility which include 2D and 3D ultrasonogram of abdomen computer assisted cholescintigraphy and 99mTc-HIDA scan.

Pallotta N [20] et al. used ultrasonogram in the assessment of gallbladder motor activity. They reported that bile flow through gallbladder can be assessed by mathematical analysis of a frequent ultrasonogram measurement of gallbladder volume even in normal and abnormal physiological condition. Although there are many advanced imaging technologies available in the primary evaluation of hepatobiliary diseases, 99mTc-HIDA scan derivatives constitute an established technique that complements morphological imaging and provides valuable information in gallbladder disease. In our study, although we did basic TUS for all patients, we used 99mTc-HIDA scan as an established technique for assessing gallbladder contractility.

H. Lambie [21] et al. explained indication of 99mTc-HIDA scintigraphy in clinical cases. They described it as a valuable diagnostic tool to assess the functional integrity of gallbladder. In our study also we found 99mTc-HIDA scintigraphy as a useful technique to demonstrate gallbladder functional integrity.

K. Riyad [19] et al. showed the role of 99mTc-HIDA scan in management of biliary pain. In patients with typical biliary pain and normal ultrasonogram, HIDA scan is a useful clinical tool. In our study we did both TUS and 99mTc-HIDA in all patients and we found 99mTc-HIDA is a very useful diagnostic tool in biliary pain patients.

Kaoutzanis C [22] et al. compared the sensitivity of ultrasonogram of abdomen and 99mTc-HIDA scan in diagnostic workup in cholecystitis patients. The sensitivity of AUS is limited to the diagnosis of acute cholecystitis in patients with upper abdominal pain. The diagnostic workup by using 99mTc-HIDA scan significantly improves sensitivity and it can be used as valuable information in the appropriate clinical setting. In our study we used TUS to diagnose the gallstone disease and to assess the size and number of stones in gallbladder. But we used 99mTc-HIDA to diagnose accurate gallbladder contractility.

De-Chuan Chan [23] et al. compared gallbladder contractility and volume characteristics in gallstone dyspepsia. Their study showed that increased gallbladder contractility after gastric distension or fatty meal might be related to dyspeptic symptoms in uncomplicated gallstone disorder. In our study all patients (100%) with good gallbladder contractility (>80% in 99mTc-HIDA scan) presented with clinical symptoms like abdominal pain, fever, and dyspepsia.

Calabuig R [24] et al. compared gallbladder dyskinesia with patients clinical symptoms like biliary colic. His results showed an abnormal emptying pattern of gallbladder was identified in patients with biliary colic. In our study gallbladder dyskinesia was compared with clinical symptoms and we found 78.8% of gallbladder dyskinesia had clinical symptoms. But it is statistically insignificant (P value-0.317) since 100% of patients with good gallbladder contractility also presented with clinical symptoms. So we cannot conclude that abnormal emptying pattern of gallbladder is the reason for the biliary colic.

Pomeianz IS [25] et al. showed no correlation of clinical symptoms and gallbladder dyskinesia. This study result is similar to our study results.

In our study most of the patients with cholelithiasis presented with some clinical symptoms (82.5%) like abdomen pain, fever, and dyspepsia and some patients presented as asymptomatic (17.5%) cholelithiasis.

Garjesh S Rai [26] et al. compared gallbladder contractility in type 2 diabetes mellitus and nondiabetic patients. Their results showed that gallbladder contractility is reduced in diabetic patients more than nondiabetic patients. But in our study there is no significant correlation between diabetics and reduced contractility (P value 0.687). Their study is a randomized study in diabetic patients irrespective of presence of gallstone, but in our study only patients with gallstones were selected. This is the probable reason for the contradicting results.

Fisher RS [27] et al. compared gallbladder emptying in patient with gallstone. Their study shows gallbladder emptying was decreased significantly in patients with gallstones compared to normal subjects.

Pomeranz IS [25] et al. also compared gallbladder emptying in a subgroup of patients with gallstones. Independently of clinical features, stone size, and number of stones, there is decreased gallbladder emptying in a subgroup of gallstone patients. In our study all patients presented with gallbladder stone. So we could compare only size of stone and number of stones with gallbladder dyskinesia.

We correlated 99mTc-HIDA scan results with the ultrasonogram of abdomen findings like

1. size of stone, less than 1cm and more than 1cm;
2. number of stones, single or multiple.

The study result shows large size stone patients (more than 1cm) significantly (P value 0.048) have reduced gallbladder contractility than small size stone patients (<1cm) in 99mTc-HIDA scan.

The study result also shows gallbladder contractility significantly reduces in multiple stone patients than single stone patients (P value 0.001)

In our study we compared histopathology report of gallbladder with ejection fraction of gallbladder. It shows in cholecystitis patients gallbladder contractility in 99mTc-HIDA scan is more significantly reduced than non-cholecystitis patients (P value 0.017).

Chang Seok Bang [28] et al. used cholescintigraphy to compare clinical relationship between cholecystitis and gallbladder contractility. Their results showed ejection fraction of gallbladder measured by cholescintigraphy could not be used for the detection of confirmation of cholecystitis. Their study result contradicts our study results. But they only correlated steatocholecystitis with cholescintigraphy irrespective of presence of gallstone in patients with cholecystitis. According to our study 99mTc-HIDA scan can be used to diagnose cholecystitis in gallstone patients.

Kautzanis C [22] et al. compared abdominal ultrasound versus 99mTc-HIDA scan in diagnosing cholecystitis. Their result shows sensitivity increased by 99mTc-HIDA scan in diagnosing cholecystitis and this result is similar to our study result.

## 7. Conclusion

Although gallbladder pathology was diagnosed with transabdominal ultrasonogram, 99mTc-HIDA scan can be used as an accurate method to diagnose the gallbladder dyskinesia. In our result, the diabetic patients did not significantly present with gallbladder dyskinesia while comparing nondiabetic patients. The relationship of gallbladder dyskinesia in symptomatic patients was not well established. But gallbladder dyskinesia in 99mTc-HIDA scan can be used to predict large size stones and multiple stones before surgery. The sensitivity can be improved by 99mTc-HIDA scan in diagnosing cholecystitis patients.

## Figures and Tables

**Table 1:** Comparison of gallbladder contraction in 99mTc-HIDA scan with various parameters of gall stone patients.

S.NO	Particulars		Gall Bladder Contraction <80% in 99mTc-HIDA Scan	Gall Bladder Contraction >80 % in 99mTc-HIDA Scan	P Value
1	Symptom of the patients	Symptomatic patients	78.8 %	100 %	0.317
		Asymptomatic patients	21.2 %	0.0 %	

2	Diabetic status of patient	Diabetic	42.4 %	28.6 %	0.681
		Non Diabetic	57.6 %	71.4 %	

3	Size of Gall stone in ultrasound	Less than and equal to 1 cm	63.6 %	100 %	0.048
		> 1cm	36.4 %	0.0 %	

4	Number of stones in ultrasound	Single	18.2 %	85.7 %	0.001
		Multiple	81.8 %	14.3 %	

5	Histopath report after laparoscopic cholecystectomy	No cholecystitis	21.2 %	71.4 %	0.017
		Cholecystitis	78.8 %	28.6 %	

## Data Availability

All data are available in our hospital records.
